# Postoperative survival in patients undergoing surgery for long bone metastases: a systematic review and Meta-analysis of outcomes across primary tumor types

**DOI:** 10.1016/j.jbo.2026.100775

**Published:** 2026-06-14

**Authors:** Tom M. de Groot, Jeroen D.R. Koning, Michelle R. Shimizu, Date van der Meij, Olivier Q. Groot, Joris J.W. Ploegmakers, Job N. Doornberg, Paul C. Jutte

**Affiliations:** a Department of Orthopaedic Surgery, University Medical Center Groningen, University of Groningen, Groningen, the Netherlands; b Department of Orthopaedic Surgery, Amsterdam Medical Centers, Amsterdam, the Netherlands; c Department of Orthopaedic Surgery, Cleveland Clinic Medical Center, Cleveland, OH, USA; d Department of Orthopaedic Surgery, Frisius Medical Center, Leeuwarden, the Netherlands; e Department of Orthopaedic Trauma Surgery, Flinders University, Flinders Medical Center, Adelaide, Australia

**Keywords:** Bone metastases, Long bone, Surgery, Survival, Systematic review, meta-analysis, Prognosis

## Abstract

**Background:**

Surgery for long bone metastases is performed to alleviate pain, restore function, and improve quality of life. Accurate postoperative survival estimates are essential for surgical planning and shared decision-making, yet no comprehensive synthesis exists across primary tumor types. This systematic review aimed to characterize postoperative survival and its temporal trend in patients undergoing surgery for long bone metastases.

**Methods:**

A systematic literature search was performed in PubMed, EMBASE, and Cochrane Library. Studies reporting survival following surgery for long bone metastases were eligible. Pooled survival estimates were computed using restricted maximum likelihood random-effects meta-analysis. Secular trend analysis used weighted linear regression with study sample size as weights. Risk of bias was assessed at the study level using the Joanna Briggs Institute (JBI) critical appraisal tools, with separate checklists applied for cohort studies and case series.

**Results:**

A total of 155 studies comprising 27,133 patients were included (1973–2024). Pooled 1-year survival was 47.9% (95% CI 44.1–51.7%, 95% prediction interval 17.7–79.7%, I^2^ = 89%; *n* = 101 studies), declining to 30.8% at 2 years and 16.9% at 5 years. Median survival was 8.0 months (IQR 6.0–11.0 months). Considerable variation was observed by tumor type: thyroid carcinoma and myeloma demonstrated the highest 1-year survival (90.0% and 70.6%, respectively), followed by breast cancer (56.4%), while lung cancer (22.7%), hepatocellular carcinoma (23.2%), and prostate cancer (26.5%) had the lowest. In the contemporary cohort (recruitment end ≥2016; *n* = 62 studies), pooled 1-year survival was 52.2% (95% CI 47.9–56.4%) and median survival was 10.0 months (IQR 7.0–13.6 months). A statistically significant positive secular trend was identified (+0.47% per year; 95% CI +0.11 to +0.82%; *p* = 0.012).

**Conclusions:**

Postoperative survival varies considerably by primary tumor type and has improved significantly over the past decade. These findings, although non-definitive and descriptive, provide contemporary long-bone metastatic disease specific survival benchmarks to inform prognostic counselling.

## Highlights

• Pooled one-year postoperative survival following surgery for long-bone metastases was 47.9%, declining to 16.9% at five years.
• Survival varies considerably by primary tumor type, ranging from 90.0% in thyroid carcinoma to 22.7% in lung cancer.
• A statistically significant positive secular trend of +0.47% per year was identified over the study period.
• In contemporary cohorts, pooled one-year survival improved to 52.2% with a median survival of 10.0 months.
• These findings provide tumor-specific survival benchmarks to inform surgical decision-making and predictive model development.

## 1 Introduction

Bone metastases occur in approximately 30% of all patients with advanced cancer, and are particularly prevalent in carcinoma of the breast, lung, and prostate, where rates of skeletal involvement reach 70–90% [1]. Among the skeletal sites affected, the long bones of the extremities are frequently involved, resulting in pain, functional impairment, and pathological fracture. In cases of severe symptoms or impending or established fracture, surgical stabilization is commonly indicated.

In contrast to spinal metastases, where surgical intervention is driven primarily by epidural cord compression or spinal instability, long bone metastases are frequently treated prophylactically — before fracture occurs — with the goals of pain relief and restoration of ambulatory function. Pathological fractures of the long bones rarely resolve without fixation, and the physical and psychological burden of an unsupported fracture in a patient with limited prognosis argues strongly for early surgical stabilization in appropriately selected patients [[2], [3]].

Two survival thresholds are considered particularly relevant to surgical planning: 90 days and 1 year. Patients with an anticipated survival shorter than 90 days may derive limited benefit from surgery or may be better served by minimally invasive palliation. Conversely, patients expected to survive beyond 1 year are candidates for more durable reconstruction [4]. Accurate preoperative survival estimation after surgical fixation of long bones is therefore fundamental to selecting appropriate treatment.

Over the past decade, multiple prognostic models have been developed to predict postoperative survival in this population, including the SORG machine learning algorithms for 90-day and 1-year mortality [[5], [6], [7]]. These models have demonstrated good performance in internal and external validation studies [[8], [9]]. However, advances in systemic oncological therapies — including targeted agents, immunotherapy, and novel hormonal therapies — have substantially altered the natural history of metastatic disease and may render survival estimates derived from older cohorts obsolete [4].

To our knowledge, no comprehensive systematic review has synthesized postoperative survival data across the breadth of primary tumor types in patients undergoing surgery for long bone metastases, nor has the secular trend in survival been formally characterized. This systematic review and pooled descriptive survival analysis aimed to: (1) provide a pooled overview of postoperative survival following surgery for long bone metastases; (2) compare survival across primary tumor types; and (3) assess whether postoperative survival has improved over time.

## 2 Methods

### 2.1 Registration and reporting

This systematic review was registered in the PROSPERO international prospective register of systematic reviews prior to study initiation (no. 1183214). Reporting follows the Preferred Reporting Items for Systematic Reviews and Meta-Analyses (PRISMA) guidelines.

Literature Search.

A systematic literature search was performed in PubMed, EMBASE, and Cochrane Library for studies published up to May 1st, 2025. Database-specific search strategies combined terms for bone metastases, long bone anatomy (femur, humerus, tibia, fibula, ulna, radius), and surgical intervention. The full search strategy is provided in Appendix 1.

### 2.2 Eligibility criteria

**Fig. 1:**
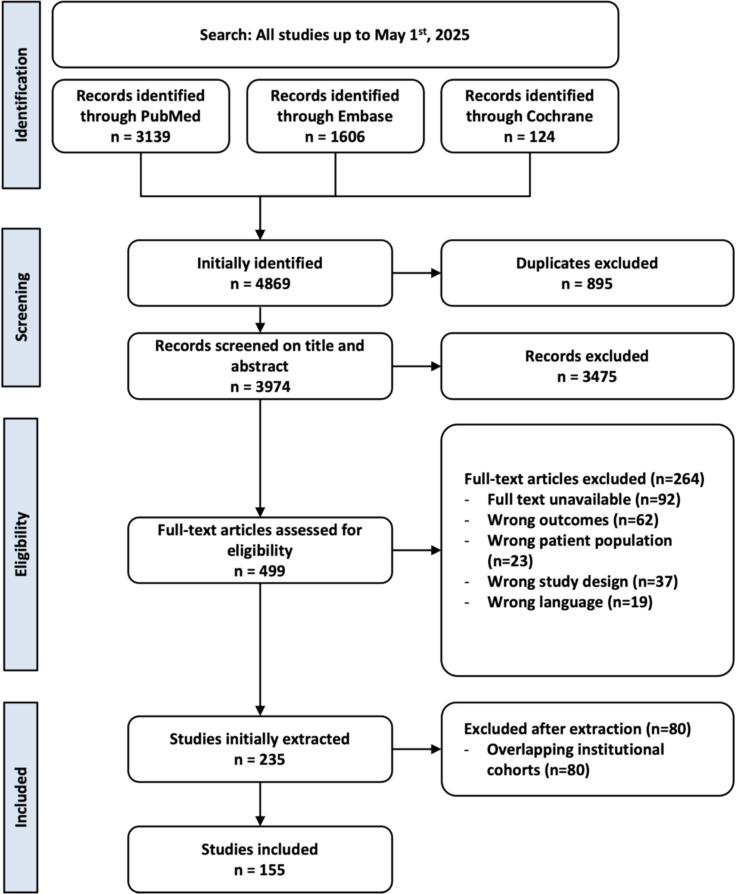
PRISMA flowchart of included studies.

Studies were included if they reported postoperative survival outcomes after surgical fixation of long bone metastases and included tumor-specific characteristics. To reduce the influence of small case series and unstable survival estimates, studies ≥10 patients were included, in line with previous work [10] Studies were excluded if they were non-English, lacked full text availability, reported on bones other than those of the extremities, reported exclusively on primary bone tumors or spinal metastases, described non-surgical management only, or were systematic or narrative reviews (Fig. 1).

### 2.3 Study selection and data extraction

Title and abstract screenings were performed independently by four reviewers (TMdG, JDRK, MRS, DvdM), with discrepancies resolved by discussion with a senior author (OQG). Full texts of potentially eligible articles were subsequently reviewed. Data were extracted on first author, year of publication, institution, journal, sample size, study design, patient demographics, primary tumor type, surgical procedure, and survival outcomes.

Survival data were extracted as reported, including: timepoint survival proportions (in-hospital, 30-day, 3-month, 1-year, 2-year, 3-year, and 5-year), median postoperative survival with measures of variability where available, and mean survival. Where studies reported Kaplan–Meier curves without numerical data, survival figures were extracted using digital extraction tools (WebPlotDigitizer 4.0). To avoid double-counting of patient cohorts, a systematic pairwise analysis of institutional affiliation and recruitment period overlap was performed across all included studies. Where confirmed overlap was identified, the study with the largest sample size was retained.

### 2.4 Risk of Bias Assessment

The methodological quality of included studies was assessed using the JBI Critical Appraisal Checklists. Studies were appraised using the checklist most appropriate to their design: cohort studies with a defined comparison group were assessed using the JBI Checklist for Cohort Studies (11 items), while case series and cohort studies without a comparison group were assessed using the JBI Checklist for Case Series (10 items). Each item was scored as Yes, No, Unclear, or Not Applicable. A summary score was calculated per study as the proportion of applicable items rated Yes. Studies were categorized as low risk of bias (≥75% of applicable items rated Yes), moderate risk (50–74%), or high risk (<50%). Risk of bias assessment was performed by two independent reviewers with discrepancies resolved by consensus.

### 2.5 Statistical analysis

Pooled timepoint survival estimates were computed using restricted maximum likelihood (REML) random-effects meta-analysis on logit-transformed proportions [11]. REML was preferred over the DerSimonian-Laird estimator for its less biased between-study variance estimates under high heterogeneity [12]. For subgroups with fewer than ten contributing studies, the Hartung-Knapp-Sidik-Jonkman correction was applied to prevent anti-conservative confidence intervals [[13], [14]]. Pooled estimates and 95% confidence intervals were back transformed to the probability scale.

To convey the clinical spread of survival estimates beyond what the confidence interval captures, 95% prediction intervals were calculated for each timepoint. Prediction intervals were not reported for subgroups with fewer than three contributing studies. Between-study heterogeneity was additionally quantified using the I^2^ statistic, representing the proportion of total variance attributable to genuine differences between studies rather than sampling error [15].

A secular trend analysis was performed by weighted linear regression of study-level 1-year survival on recruitment end year, with study sample size as weights, yielding a slope estimate with 95% confidence interval. Sensitivity analyses examined the robustness of the secular trend to the choice of time reference, repeating the regression using study start year and study midpoint year as alternative predictors (Supplementary Table S2).

Subgroup analyses were conducted by primary tumor type, risk of bias, and inclusion period.

The potential for reporting bias was assessed by visual inspection of funnel plot asymmetry for the primary outcome of one-year survival, supplemented by Egger's test. Given the substantial heterogeneity observed across studies, funnel plot asymmetry was interpreted cautiously, as asymmetry in this context may reflect genuine between-study differences in population or treatment characteristics rather than selective non-publication of unfavorable results.

The overall certainty of the evidence was assessed narratively in accordance with GRADE principles. Given the predominance of retrospective observational study designs, the high between-study heterogeneity (I^2^ = 89%), and the recognized risk of selection bias inherent to surgical cohorts, the certainty of the pooled estimates was considered low to moderate. These assessments are reflected in the interpretation of findings and the framing of clinical recommendations.

All statistical analyses were performed in Python (version 3.12) using the NumPy, SciPy, and Pandas libraries. Statistical significance was defined as *p* < 0.05.

### 2.6 Data availability

The data extraction template, study-level data underlying all analyses, as well as the analytical code are available from the corresponding author upon reasonable request. No individual patient data were used or generated in this review.

## 3 Results

### 3.1 Study selection and characteristics

A total of 155 studies were included, comprising 27,133 patients. Publication years ranged from 1973 to 2024, with 73% of studies published from 2010 onwards. Most studies were retrospective cohort studies (*n* = 104, 67%) or case series (*n* = 43, 28%). The majority were single-center (*n* = 112, 72%) studies and enrolled a general mixed-tumor population without specific primary tumor type (*n* = 144, 93%). The median sample size was 81 patients (IQR 38–158; range 10–1920). European institutions contributed the largest proportion of studies (*n* = 63, 41%), followed by North America (*n* = 46, 30%) and Asia and the Middle East (*n* = 37, 24%). The femur was the most investigated site (*n* = 71, 46% in isolation; *n* = 55, 36% in combination with other long bones), and multiple fixation types were reported in 46% of studies, reflecting heterogeneous surgical populations. Postoperative survival was the primary outcome in 83 studies (54%) and a secondary outcome in a further 63 (41%). Full characteristics are presented in Table 1.

**Table 1:** Characteristics of included studies (*n* = 155).

Characteristic	n (%)
Studies included	155 (100%)
Total patients (excl. Registry studies)	27,133
Median sample size per study (IQR; range)	81 (38–158; 10–1920)

Publication year	
1970–1979	1 (1%)
1980–1989	0 (0%)
1990–1999	11 (7%)
2000–2009	30 (19%)
2010–2019	60 (39%)
2020–present	52 (34%)

Geographic region	
Europe	63 (41%)
North America	46 (30%)
Asia / Middle East	37 (24%)
Oceania	0 (0%)
Latin America	2 (1%)
Multi-national	7 (5%)

Study design	
Retrospective cohort study	104 (67%)
Case series	43 (28%)
Prospective cohort study	4 (3%)
Registry study	2 (1%)
Randomized controlled trial	1 (1%)

Setting	
Single-center	112 (72%)
Multi-center	41 (26%)
Not reported	2 (1%)

Patient population	
General (mixed tumor types)	144 (93%)
Single tumor type	10 (6%)
Not reported	1 (1%)

Tumor locations investigated	
Femur only	71 (46%)
Humerus only	24 (15%)
Femur + Humerus	12 (8%)
Multiple long bones	43 (28%)
Not reported	5 (3%)

Surgical intervention	
Intramedullary nail	30 (19%)
Endoprosthetic reconstruction	30 (19%)
Plate-screw fixation	3 (2%)
Multiple fixation types	71 (46%)
Other / mixed	18 (12%)
Not reported	3 (2%)

Primary outcome	
Survival / mortality	83 (54%)
Functional outcome	37 (24%)
Post-operative complications	25 (16%)
Implant survival	0 (0%)
Quality of life	2 (1%)
Other	6 (4%)
Not reported	2 (1%)

Secondary outcome (most common)	
Survival / mortality	63 (41%)
Post-operative complications	45 (29%)
Functional outcome	23 (15%)
Implant survival	1 (1%)
Other	8 (5%)
Not reported	15 (10%)

Two large administrative registry studies excluded from all quantitative analyses: Behnke 2017 (*n* = 124,904) and Christ 2023 (*n* = 407,893). Total patients and sample size statistics refer to the remaining 155 included studies.

### 3.2 Postoperative survival across all studies

Pooled 1-year survival across all studies was 47.9% (95% CI 44.1–51.7%; 95% prediction interval 17.7–79.7%; I^2^ = 89%; *n* = 101 studies), with considerable variation across individual studies (range 10.0–96.1%). Survival declined progressively at later timepoints: pooled 3-month survival was 74.4% (95% CI 70.4–78.0%; *n* = 49 studies), 6-month survival was 64.9% (95% CI 60.3–69.2%; *n* = 61 studies), 2-year survival was 30.8% (95% CI 25.8–36.3%; *n* = 64 studies), and 5-year survival was 16.9% (95% CI 13.1–21.4%; *n* = 29 studies). High I^2^ values (86–92%) were observed across all timepoints, indicating substantial heterogeneity across studies (Table 2). Median survival data were available from 41 studies; the median of reported medians was 8.0 months (IQR 6.0–11.0 months; range 2.5–140.0 months).

**Table 2:** Pooled postoperative survival — general surgical population (all studies).

Timepoint	n	Pooled survival	95% CI	95% PI	I^2^	Range
in-hospital	8	94.3%	89.3–97%§	74.5–98.9%	45%	86.1–98%
1-month	36	92.4%	90.1–94.2%	72.2–98.3%	89%	69.5–100%
3-month	49	74.4%	70.4–78%	43.7–91.6%	88%	24–100%
6-month	61	64.9%	60.3–69.2%	30.8–88.4%	86%	18–97%
1-year	101	47.9%	44.1–51.7%	17.7–79.7%	89%	10–96.1%
2-year	64	30.8%	25.8–36.3%	6.2–74.8%	92%	0–95%
3-year	43	24.8%	20.4–29.9%	6.1–62.6%	89%	4.1–95%
5-year	29	16.9%	13.1–21.4%	4.2–48.7%	89%	1.1–47.9%
**Median survival**	41	8 months	6–11 months	–	–	2.5–140 months

REML = restricted maximum likelihood random-effects meta-analysis on logit-transformed proportions. § REML+HKSJ = REML with Hartung-Knapp-Sidik-Jonkman correction applied (t-distribution with k − 1 degrees of freedom); used for subgroups with k < 10 to account for uncertainty in τ^2^ estimation. 95% CI = confidence interval on pooled mean. 95% PI = 95% prediction interval, representing the range within which the true survival of a new study from the same population would be expected to fall with 95% probability; computed on t(k − 2) distribution. ‡ PI not calculable for k < 3. † Single study; no pooled estimate.

### 3.3 Postoperative survival by primary tumor type

One-year survival stratified by primary tumor type is presented in Table 3. Thyroid carcinoma and myeloma demonstrated the most favorable 1-year survival (90.0%, 95% CI 90.0–90.0%, *n* = 2 studies; and 70.6%, 95% CI 63.9–76.4%, n = 2 studies, respectively), followed by breast cancer (56.4%; 95% CI 46.2–66.0%; *n* = 16 studies) and renal cell carcinoma (42.0%; 95% CI 23.5–63.1%; *n* = 8 studies). Lung cancer (22.7%; 95% CI 12.2–38.5%; *n* = 13 studies), hepatocellular carcinoma (23.2%; 95% CI 5.3–62.0%; *n* = 2 studies), and prostate cancer (26.5%; 95% CI 14.3–43.9%; n = 8 studies) showed the lowest 1-year survival.

**Table 3:** Pooled 1-year postoperative survival by primary tumor type — all studies.

Primary tumor type	n	Pooled 1-year survival	95% CI	95% PI	I^2^	Range
General population	101	47.7%	43.9–51.5%	17.9–79.2%	88%	10–96.1%
Breast	16	56.4%	46.2–66%	18.5–88%	92%	19–81%
Lung	13	22.7%	12.2–38.5%	1.5–85%	95%	0–67%
Renal cell	8	42%	23.5–63.1%	5.3–90.4%	93%	9.1–72%
Prostate	8	26.5%	14.3–43.9%	3.8–76.8%	92%	9–60%
Myeloma	2	70.6%	63.9–76.4%	n/a	0%	70–71%
Thyroid	2	90%	90–90%	n/a	0%	90–90%
Hepatocellular	2	23.2%	0–100%	n/a	89%	12–43.3%
Sarcoma	1	45%	–	n/a	–	45–45%

REML = restricted maximum likelihood random-effects meta-analysis on logit-transformed proportions. § REML+HKSJ = REML with Hartung-Knapp-Sidik-Jonkman correction applied (t-distribution with k − 1 degrees of freedom); used for subgroups with k < 10 to account for uncertainty in τ^2^ estimation. 95% CI = confidence interval on pooled mean. 95% PI = 95% prediction interval, representing the range within which the true survival of a new study from the same population would be expected to fall with 95% probability; computed on t(k − 2) distribution.

Thyroid carcinoma had the longest median survival (25.0 months; IQR 23.9–26.2 months; n = 2 studies), followed by myeloma (14.8 months; IQR 9.0–20.5 months; n = 2 studies), breast cancer (12.8 months; IQR 8.0–22.0 months; *n* = 9 studies), and renal cell carcinoma (10.7 months; IQR 7.1–13.0 months; *n* = 7 studies). Lung cancer had the shortest median survival (4.0 months; IQR 3.1–4.2 months; n = 7 studies), followed by prostate cancer (5.8 months; IQR 4.8–6.8 months; *n* = 6 studies; Table 4).

**Table 4:** Median postoperative survival by primary tumor type — all studies.

Primary tumor type	n	Median survival	IQR	Range
General population	41	8 months	6–11 months	2.5–140 months
Breast	9	12.8 months	8–22 months	3.3–26.5 months
Lung	7	4 months	3.1–4.2 months	3–6.5 months
Renal cell	7	10.7 months	7.1–13 months	5.7–140 months
Prostate	6	5.8 months	4.8–6.8 months	1.6–11.1 months
Myeloma	2	14.8 months	9–20.5 months	3.3–26.3 months
Thyroid	2	25 months	23.9–26.2 months	22.7–27.4 months
Sarcoma	1	11 months	–	–
Lymphoma	1	17.6 months	–	–

Median of study-level reported medians. Studies reporting mean survival only are excluded. Single-study entries have no IQR or range.

### 3.4 Secular trend and contemporary cohorts

**Fig. 2:**
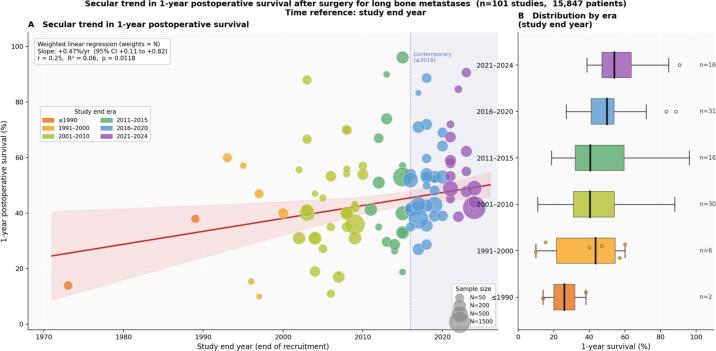
**Secular trend in 1-year postoperative survival after surgery for long bone metastases, by study end year.** Panel A shows the weighted linear regression of study-level 1-year survival against recruitment end year, with bubble size proportional to study sample size (N). The red line represents the weighted least squares regression line (weights = N) with 95% confidence band. The blue dashed line and shaded region denote the contemporary cohort boundary (recruitment end ≥2016). Era colours correspond to the study end year groupings shown in the legend. Weighted linear regression: slope + 0.47%/yr (95% CI +0.11 to +0.82%); *r* = 0.25; R^2^ = 0.062; *p* = 0.012; *n* = 101 studies. Panel B shows the distribution of study-level 1-year survival by era using box-and-whisker plots; horizontal lines within boxes indicate medians, box limits indicate interquartile range, whiskers extend to 1.5 × IQR, and outliers are shown as individual points. (For interpretation of the references to colour in this figure legend, the reader is referred to the web version of this article.)

A statistically significant positive secular trend in 1-year survival was identified over the study period (weighted linear regression, weights = N: +0.47% per year; 95% CI +0.11 to +0.82%; *r* = 0.25; R^2^ = 0.062; *p* = 0.012; *n* = 101 studies) (Fig. 2). This trend was consistent across all time references examined (study end year, midpoint year, and start year), with slopes ranging from +0.25% to +0.47% per year and remaining statistically significant in each case (Supplementary Table S2). The modest R^2^ of 0.062 indicates that calendar time explains only a small proportion of the variance in reported survival.

A secondary analysis was restricted to studies with recruitment periods ending in 2016 or later (*n* = 62 studies), reflecting contemporary surgical practice. In this contemporary cohort, pooled 1-year survival was 52.2% (95% CI 47.9–56.4%; 95% PI 26.1–77.1%; I^2^ = 87%; *n* = 47 studies), and median survival was 10.0 months (IQR 7.0–13.6 months; *n* = 19 studies). Pooled 3-month and 6-month survival in the contemporary cohort were 77.5% (95% CI 71.2–82.8%; *n* = 25 studies) and 71.0% (95% CI 64.3–76.8%; n = 25 studies), respectively. Among tumor types in the contemporary cohort, breast cancer demonstrated the highest 1-year survival (73.5%; 95% CI 64.2–81.1%; *n* = 5 studies). Although significant, these modest secular increases in postoperative survival are not indicative of any specific surgical causality. Other estimates in the contemporary cohort should be interpreted with caution given the small number of contributing studies (Table 5, Table 6).

**Table 5:** Pooled postoperative survival — contemporary cohort (recruitment end ≥2016, n = 62 studies).

Timepoint	n	Pooled survival	95% CI^§^	95% PI	I^2^	Range
1-month	20	91.2%	88–93.5%	71.5–97.7%	87%	69.5–100%
3-month	25	77.5%	71.2–82.8%	38.7–95%	92%	24–95.2%
6-month	25	71%	64.3–76.8%	34.5–91.9%	89%	45–97%
1-year	47	52.2%	47.9–56.4%	26.1–77.1%	87%	27–90.6%
2-year	23	37.3%	28.3–47.3%	7–82.4%	92%	11–95%
3-year	19	29.8%	21.4–39.8%	5.2–76.7%	90%	10.7–95%
5-year	15	17.4%	11.9–24.7%	3.2–57%	90%	2.7–47.9%
**Median survival**	19	10 months	7–13.6 months	–	–	4.7–140 months

REML = restricted maximum likelihood random-effects meta-analysis on logit-transformed proportions. 95% CI = confidence interval on pooled mean. 95% PI = 95% prediction interval, representing the range within which the true survival of a new study from the same population would be expected to fall with 95% probability.

**Table 6:** Pooled 1-year postoperative survival by primary tumor type — contemporary cohort (recruitment end ≥2016).

Primary tumor type	n	Pooled 1-year survival	95% CI^§^	95% PI	I^2^	Range
General population	47	51.7%	47.6–55.8%	27–75.6%	86%	27–90.6%
Breast	5	73.5%	64.2–81.1%	47.8–89.4%	71%	64.1–81%
Lung	4	37%	7.7–80.5%	0.2–99.5%	97%	16.7–66.6%
Renal cell	2	24.3%	0–100%	–	98%	9.1–50%
Prostate	2	29.6%	0.1–99.7%	–	96%	20–41.7%
Myeloma	1	70%†	–	–	–	70–70%

REML = restricted maximum likelihood random-effects meta-analysis on logit-transformed proportions. ^§^REML+HKSJ = REML with Hartung-Knapp-Sidik-Jonkman correction applied (t-distribution with k − 1 degrees of freedom); used for subgroups with k < 10 to account for uncertainty in τ^2^ estimation. 95% CI = confidence interval on pooled mean. 95% PI = 95% prediction interval, representing the range within which the true survival of a new study from the same population would be expected to fall with 95% probability; † Single study; no pooled estimate.

Risk of Bias.

Seventy-seven studies were appraised using the JBI Case Series Checklist, with a mean summary score of 67.9% (SD 22.4%; range 0–100%); 47% (*n* = 36 studies) were classified as low (≥75%), 39% (*n* = 30 studies) as moderate, and 14% (*n* = 11 studies) as high risk of bias. Strengths across studies included clear reporting of inclusion criteria (84%), demographics (82%), and outcomes (84%). The most prevalent weaknesses were insufficient reporting of clinical information (42% rated No) and uncertainty regarding consecutive participant inclusion (Unclear in 43% of studies).

Seventy-nine studies were appraised using the JBI Cohort Study Checklist, with a mean summary score of 70.0% (SD 20.8%; range 18–100%); 41% (*n* = 32 studies) were low, 43% (*n* = 34 studies) were moderate, and 16% (*n* = 13 studies) were high risk. Consistent strengths included confirmation that participants were free from the outcome at baseline (96%), validity of exposure measurement (94%), and adequacy of follow-up (85%). The most prominent limitation was infrequent reporting of strategies to address confounding, with only 37% of applicable items rated Yes (Supplementary Tables S3 and S4).

### 3.5 Risk of bias sensitivity analysis

To assess whether pooled survival estimates were influenced by methodological quality, all primary analyses were repeated exclusively for studies rated as low risk of bias (JBI score ≥ 75%; *n* = 68 studies). Pooled timepoint survival estimates were highly consistent between the full cohort and the low risk of bias subgroup across all timepoints, with differences of less than 3 percentage points at every interval. Median survival was identical in both analyses at 8.0 months. Tumor-type specific 1-year survival estimates were similarly robust. The secular trend analysis yielded a statistically significant positive slope in both the full cohort (+0.44%/yr; 95% CI +0.09 to +0.80%; *p* = 0.016; *n* = 100 studies) and the low risk of bias sub cohort (+0.71%/yr; 95% CI +0.08 to +1.33%; *p* = 0.032; *n* = 47 studies), with the slope modestly larger in the latter, suggesting that the temporal improvement in survival is not an artefact of lower-quality studies (Supplementary Table S5).

## 4 Discussion

This systematic review and pooled descriptive survival analysis synthesized postoperative survival data from 155 studies comprising 27,133 patients undergoing surgery for long bone metastases. The key findings are: (1) pooled 1-year, 2-year, and 5-year survivals was approximately 48%, 31%,and 17%, respectively; (2) survival rates varied substantially by primary tumor type, with thyroid carcinoma and myeloma having the most favorable prognosis and lung cancer and hepatocellular carcinoma the least; (3) there was a significant secular improvement in survival of +0.47% per year; and (4) contemporary cohort analysis demonstrated a pooled 1-year survival of 52.2% and a median of 10.0 months.

The postoperative 1-year survival of approximately 48% for the entire cohort aligns with survival thresholds used clinically to guide surgical decision-making. Current practice guidelines generally consider patients with a prolonged survival as candidates for more durable reconstruction that outlives the patient, such as endoprosthetic replacement [16]. For patients in whom survival cannot be reliably predicted to exceed 1 year, the additional surgical burden of endoprosthetic reconstruction is difficult to justify, and intramedullary fixation represents the appropriate default. However, the substantial variation in survival by primary tumor type underscores that population-level estimates should not replace individualized prognostic assessment. For patients with favorable tumor biology and adequate performance status, more durable reconstruction remains appropriate and the risk of under-treating a long-term survivor is a clinical error of equal consequence.

The marked differences in survival by tumor type are clinically important and largely consistent with the known natural history of these diseases. Myeloma and thyroid carcinoma, both characterized by relatively effective systemic therapies and often indolent disease courses, demonstrated the highest 1-year survival rates. Breast cancer also showed favorable survival (56.4%), consistent with the impact of hormonal therapies and CDK4/6 inhibitors on disease trajectory [17]. In contrast, lung cancer — characterized by rapid progression and, in the pre-immunotherapy era, limited therapeutic options [18]— showed the lowest survival among the higher-volume subgroups (22.7%), which is concordant with clinical experience. Renal cell carcinoma occupied an intermediate position (median 10.7 months across all studies; 12.9 months in the contemporary cohort), reflecting its variable clinical course and responsiveness to targeted agents such as sunitinib and pazopanib [19]. However, these findings should be interpreted with caution given the small number of contributing studies for most tumor-type subgroups and the wide prediction intervals. Accordingly, all tumor-specific estimates should be regarded as low-certainty evidence. Wide prediction intervals reflect meaningful variation in patient selection, disease burden, treatment era, and surgical indication that cannot be accounted for in a descriptive pooled analysis. Tumor-specific estimates should be regarded as preliminary benchmarks, and dedicated studies with prospective tumor-specific designs are needed to confirm these observations.

Prostate cancer demonstrated unexpectedly low 1-year survival in this analysis (26.5%, 95% CI 14.3–43.9%), which warrants contextualization. The majority of contributing studies recruited before the routine availability of enzalutamide and abiraterone: van Doorn et al. recruited through 1995, Forsberg et al. and Willeumier et al. through 2011 and 2015 respectively, and Meynard et al. through 2017. The two most contemporary studies reported considerably higher survival — Mehnert et al. (2024) 41.7% and Mohamed-Haflah et al. (2017) 60% — consistent with a meaningful improvement in the modern treatment era. The pooled prostate cancer estimate should therefore be interpreted as reflecting predominantly historical populations and likely underestimates survival under current systemic therapy protocols.

The secular improvement of +0.47% per year in 1-year survival across the study period ismodest and probably not attributable to any surgical causation…It is more indicative of the cumulative impact of numerous incremental advances in oncological treatment over the last decades. Given the heterogeneous mix of primary tumor types, any survival gain achieved within individual malignancies are expected to be diluted when only the post-operative subgroups fit for surgery are analyzed in this review. Consequently, a slight secular improvement in postoperative survival may be indicative of broader survival increase in multiple primary tumor categories. The approval of immune checkpoint inhibitors for non-small cell lung cancer (2015–2016), CDK4/6 inhibitors for breast cancer (2015), and second-generation androgen receptor pathway inhibitors for prostate cancer have each transformed the survival landscape for patients with metastatic bone disease [[17], [20], [21]]. This improvement was also seen when analysis was restricted to exclusively studies with low risk of bias (+0.71% per year), arguing against the possibility that the observed improvement is an artefact of methodological characteristics of lower-quality studies. This temporal trend has important implications for predictive modelling: algorithms trained on historical cohorts are likely to be prone to systematic underestimation of current survival trends, and contemporaneous training data are needed for a more accurate prognostication.

The high I^2^ values observed across all analyses (86–95%) are notable but not surprising. This review spans over five decades of surgical practice, encompasses patients with markedly different primary tumors, and includes studies ranging from small single-institution case series to large multicenter cohorts. The use of REML rather than the DerSimonian-Laird estimator reduces bias in τ^2^ estimation, and the HKSJ correction ensures that confidence intervals appropriately reflect uncertainty when few studies contribute. Nevertheless, absolute pooled estimates should be interpreted as descriptive summaries of the literature rather than precise effect estimates. The 95% prediction intervals (which for 1-year survival ranged from 17.7 to 79.7% across all studies) are the most clinically meaningful uncertainty estimates, as they reflect the true range of survival that might be expected in a new cohort from the same population and are substantially wider than the confidence intervals on the pooled mean.

### 4.1 Limitations

Several limitations of this review merit consideration. First, inclusion criteria varied considerably across studies, leading to significant heterogeneity in primary tumor types, extent of metastatic disease, performance status, comorbidity burden, and surgical indications investigated in all studies. Studies varied in whether they included all patients undergoing surgery or restricted inclusion to specific procedures, and in whether prophylactic fixation was included alongside treatment of completed fractures. These sources of clinical heterogeneity likely contributed to the substantial interstudy variance observed across all analyses and should be considered when interpreting the pooled estimates.

Second, all survival data were extracted at the study level without access to individual patient data. This precluded adjustment for key prognostic covariates or reconstruction of full Kaplan-Meier curves for all studies. Where studies reported survival graphically rather than numerically, values were extracted by visual inspection of Kaplan-Meier curves using digital extraction tools, introducing a degree of measurement error that is difficult to quantify but unlikely to be systematic.

Third, surgical selection bias comprises a limitation. The included cohorts comprise patients that underwent operative treatment exclusively. With surgical decision-making influenced by patient fitness, disease burden and estimated survival. Along with local practice patterns and surgical preference for each reference that was included. Patients analyzed in this review therefore represent a more favorable subset of the metastatic population. As patients with limited life expectancy or poor performance status may be managed conservatively or non-operatively. The results of this review should be interpreted in the context of patients that are evaluated for surgery, and their postoperative results. Not merely a reflection of full metastatic population.

Fourth, publication bias is a plausible concern. Studies reporting poor outcomes or high perioperative mortality may be less likely to be published, and smaller single-center series may disproportionately represent institutions with surgical expertise or favorable patient selection. The secular trend analysis demonstrated attenuation of the slope when weighted by sample size compared to unweighted estimates, which is consistent with smaller studies tending to report higher survival. Funnel plot asymmetry was not formally assessed given the insufficient number of studies in most tumor-specific subgroups.

Fifth, the secular trend analysis is subject to inherent limitations. Study end year is an imprecise proxy for treatment era: studies with long recruitment windows spanning multiple treatment eras are represented by a single time point, and the analysis cannot account for within-era variation in systemic therapy, surgical technique, or perioperative care. The modest R^2^ of 0.062 confirms that calendar time explains only a small fraction of the variance in reported survival, and the trend should be interpreted as a broad signal of improvement rather than a precise quantification of temporal change. Causal attribution to any specific therapeutic advance is not possible from aggregate study-level data.

Sixth, tumor-specific survival estimates are based on a limited number of studies for most subgroups, ranging from two studies for thyroid and hepatocellular carcinoma to 16 for breast cancer. The wide confidence intervals and residual heterogeneity within subgroups reflect meaningful variation in patient selection and treatment context that cannot be resolved without dedicated prospective tumor-specific studies. Future research should prioritize adequate and standardized reporting of survival outcomes stratified by tumor type, as inconsistent or incomplete reporting across studies significantly limits the ability to pool data in meaningful synthesis.

Seventh, data on the extent of extra-skeletal and visceral metastatic disease were not available at the study level. Because overall survival in metastatic cancer is influenced strongly by total systemic disease burden, the present analysis cannot isolate the prognostic contribution of bone metastases from that of concurrent visceral disease. Survival is instead characterized according to primary tumor type, distilling the prognostic signal associated with the tumor of origin in patients selected for surgery, and the resulting estimates should be interpreted with this framing in mind.

## 5 Conclusion

Postoperative survival following surgery for long bone metastases varies substantially by primary tumor type and has improved significantly over recent decades. Pooled contemporary 1-year survival is approximately 52%, with a median of 10 months. Thyroid carcinoma, myeloma, and breast cancer confer the most favorable prognosis, while lung cancer and hepatocellular carcinoma are associated with the shortest survival. These findings provide updated survival benchmarks that can inform preoperative counselling, surgical decision-making, and the development and validation of contemporary prognostic models. The wide prediction intervals underscore the need for prospective collaborative registries with standardized data collection to generate more precise tumor-specific estimates. These conclusions derive from predominantly retrospective, observational evidence of low to moderate certainty and should be interpreted accordingly.

Declaration of generative AI and AI-assisted technologies in the manuscript preparation process.

During the preparation of this work the author(s) used Claude Sonnet 4.6 (Anthropic) in order to improve readability. After using this tool/service, the author(s) reviewed and edited the content as needed and take(s) full responsibility for the content of the published article.

## Funding

This study received no specific funding from public, commercial, or not-for-profit funding agencies.

## CRediT authorship contribution statement

**Tom M. de Groot:** Writing – review & editing, Writing – original draft, Methodology, Formal analysis, Data curation, Conceptualization. **Jeroen D.R. Koning:** Writing – review & editing, Writing – original draft, Data curation. **Michelle R. Shimizu:** Writing – review & editing, Writing – original draft, Data curation. **Date van der Meij:** Writing – review & editing, Writing – original draft, Data curation. **Olivier Q. Groot:** Writing – review & editing, Supervision, Data curation, Conceptualization. **Joris J.W. Ploegmakers:** Writing – review & editing, Supervision. **Job N. Doornberg:** Writing – review & editing, Supervision. **Paul C. Jutte:** Writing – review & editing, Supervision, Conceptualization.

## Declaration of competing interest

The authors declare that they have no known competing financial interests or personal relationships that could have appeared to influence the work reported in this paper.

## Appendix A Search Strategies

The following database-specific search strategies were used to identify eligible studies. Searches were conducted in PubMed, Embase, and the Cochrane Central Register of Controlled Trials.

1. PubMed.

(“bone metastasis”[tiab] OR “bone metastases”[tiab] OR “skeletal metastasis”[tiab].

OR “skeletal metastases”[tiab] OR “metastatic bone disease”[tiab].

OR “Bone Neoplasms/secondary”[Mesh]).

AND

(femur[tiab] OR femoral[tiab] OR humerus[tiab] OR humeral[tiab].

OR tibia[tiab] OR tibial[tiab] OR fibula[tiab] OR fibular[tiab].

OR ulna[tiab] OR ulnar[tiab] OR radius[tiab] OR radial[tiab].

OR “long bone”[tiab] OR “long bones”[tiab] OR “extremity”[tiab].

OR “extremities”[tiab] OR “appendicular skeleton”[tiab]).

AND

(surgery[tiab] OR surgical[tiab] OR resection[tiab] OR resect*[tiab].

OR fixation[tiab] OR curettage[tiab] OR cement*[tiab].

OR arthroplasty[tiab] OR “joint replacement”[tiab] OR endoprosthe*[tiab].

OR “surgical management”[tiab] OR “limb salvage”[tiab].

OR “orthopaedic surgery”[tiab] OR “orthopaedic surgery”[tiab].

OR “Surgical Procedures, Operative”[Mesh]).

2. Embase.

(‘bone metastasis’:ti,ab OR ‘bone metastases’:ti,ab OR ‘skeletal metastasis’:ti,ab.

OR ‘skeletal metastases’:ti,ab OR ‘metastatic bone disease’:ti,ab.

OR ‘bone neoplasm’/exp./mj AND ‘secondary’/exp.).

AND

(femur:ti,ab OR femoral:ti,ab OR humerus:ti,ab OR humeral:ti,ab.

OR tibia:ti,ab OR tibial:ti,ab OR fibula:ti,ab OR fibular:ti,ab.

OR ulna:ti,ab OR ulnar:ti,ab OR radius:ti,ab OR radial:ti,ab.

OR ‘long bone’:ti,ab OR ‘long bones’:ti,ab OR extremity:ti,ab.

OR extremities:ti,ab OR ‘appendicular skeleton’:ti,ab).

AND

(surgery:ti,ab OR surgical:ti,ab OR resection:ti,ab OR resect*:ti,ab.

OR fixation:ti,ab OR curettage:ti,ab OR cement*:ti,ab.

OR arthroplasty:ti,ab OR ‘joint replacement’:ti,ab OR endoprosthe*:ti,ab.

OR ‘surgical management’:ti,ab OR ‘limb salvage’:ti,ab.

OR ‘orthopaedic surgery’:ti,ab OR ‘orthopaedic surgery’:ti,ab.

OR ‘surgical procedure’/exp.).

3. Cochrane Central.

(“bone metastasis” OR “bone metastases” OR “skeletal metastasis”.

OR “skeletal metastases” OR “metastatic bone disease”):ti,ab,kw.

AND

(femur OR femoral OR humerus OR humeral OR tibia OR tibial.

OR fibula OR fibular OR ulna OR ulnar OR radius OR radial.

OR “long bone” OR “long bones” OR extremity OR extremities.

OR “appendicular skeleton”):ti,ab,kw.

AND

(surgery OR surgical OR resection OR fixation OR curettage OR cement*.

OR arthroplasty OR “joint replacement” OR endoprosthe*.

OR “surgical management” OR “limb salvage”.

OR “orthopaedic surgery” OR “orthopaedic surgery”):ti,ab,kw.

## Appendix B Supplementary data

Supplementary Table 1. Median postoperative survival by primary tumor type - Contemporary cohort (recruitment end >2016) Supplementary Table 2. Secular trend sensitivity analysis by time reference (weighted linear regression, weights = N)
