# Supplementary material for: Postoperative survival in patients undergoing surgery for long bone metastases: a systematic review and Meta-analysis of outcomes across primary tumor types
Source: J Bone Oncol. 2026 Jun 14;59:100775. doi: 10.1016/j.jbo.2026.100775 (PMC13320406; doi:10.1016/j.jbo.2026.100775)
Supplement: Supplementary file 1 — Supplementary Table 1. Median postoperative survival by primary tumor type - Contemporary cohort (recruitment end >2016) Supplementary Table 2. Secular trend sensitivity analysis by time reference (weighted linear regression, weights = N) [file mmc1.docx]

**Supplementary Table S1.** Median postoperative survival by primary tumor type — contemporary cohort (recruitment end ≥2016)

| **Primary tumor type** | **n** | **Median survival** | **IQR** | **Range** |
| --- | --- | --- | --- | --- |
| General population | 19 | 10 months | 7–13.6 months | 4.7–140 months |
| Breast | 4 | 23 months | 20.2–24.6 months | 15–26.5 months |
| Lung | 4 | 4.1 months | 3.8–4.8 months | 3–6.5 months |
| Renal cell | 4 | 13 months | 11.7–44.8 months | 8.2–140 months |
| Prostate | 3 | 5.7 months | 5.1–8.4 months | 4.5–11.1 months |

*Median of study-level reported medians within the contemporary cohort.*

**Supplementary Table S2.** Secular trend sensitivity analysis by time reference (weighted linear regression, weights = N)

| **Time reference** | **n** | **Slope** | **95% CI** | **r** | **R²** | **p** |
| --- | --- | --- | --- | --- | --- | --- |
| Study end year | 101 | +0.47%/yr | 0.11 to 0.82 | 0.25 | 0.062 | 0.0118 |
| Study midpoint year | 101 | +0.37%/yr | 0.06 to 0.67 | 0.23 | 0.053 | 0.0209 |
| Study start year | 101 | +0.25%/yr | 0 to 0.49 | 0.20 | 0.038 | 0.0497 |

*All three time references yield a statistically significant positive slope. Study end year is the primary pre-specified reference.*

**Supplementary Table S3.** Risk of bias — JBI Case Series Checklist (n = 77)

| **Domain** | **Yes** | **No** | **Unclear** | **NR** |
| --- | --- | --- | --- | --- |
| Inclusion criteria clearly stated | 65 (84%) | 5 (6%) | 6 (8%) | — |
| Condition measured in standard way | 46 (60%) | 5 (6%) | 25 (32%) | — |
| Valid methods for case identification | 47 (61%) | 4 (5%) | 25 (32%) | — |
| Consecutive inclusion of participants | 34 (44%) | 9 (12%) | 33 (43%) | — |
| Complete inclusion of participants | 42 (55%) | 5 (6%) | 28 (36%) | 1 |
| Demographics clearly reported | 63 (82%) | 11 (14%) | 2 (3%) | — |
| Clinical information clearly reported | 37 (48%) | 32 (42%) | 6 (8%) | 1 |
| Outcomes / follow-up clearly reported | 65 (84%) | 9 (12%) | 2 (3%) | — |
| Site or clinic demographics reported | 64 (83%) | 9 (12%) | 3 (4%) | — |
| Appropriate statistical analysis used | 58 (75%) | 15 (19%) | 3 (4%) | — |
| **Overall: 36/77 (47%) scored ≥75%. Mean 67.9% (SD 22.4%; range 0–100%). 11 studies <50%, 30 50–74%, 36 ≥75%.** | | | | |

*JBI Critical Appraisal Checklist for Case Series (10 items). Applied to 77 studies. Percentages out of n=77. NR = not reported.*

**Supplementary Table S4.** Risk of bias — JBI Cohort Study Checklist (n = 79)

| **Domain** | **Yes** | **No** | **Unclear** | **N/A** | **NR** |
| --- | --- | --- | --- | --- | --- |
| Groups similar and from same population | 43 (54%) | 6 (8%) | 13 (16%) | — | — |
| Exposures measured similarly between groups | 58 (73%) | 2 (3%) | 4 (5%) | — | — |
| Exposure measured in valid and reliable way | 74 (94%) | 1 (1%) | 2 (3%) | — | — |
| Confounding factors identified | 50 (63%) | 11 (14%) | 16 (20%) | — | 2 |
| Strategies to address confounding stated | 29 (37%) | 48 (61%) | 1 (1%) | — | — |
| Participants outcome-free at study start | 76 (96%) | 0 (0%) | 3 (4%) | — | — |
| Outcomes measured in valid and reliable way | 57 (72%) | 3 (4%) | 19 (24%) | — | — |
| Follow-up time reported and sufficient | 67 (85%) | 6 (8%) | 6 (8%) | — | — |
| Follow-up completeness addressed | 35 (44%) | 6 (8%) | 37 (47%) | — | — |
| Strategies for incomplete follow-up stated | 54 (68%) | 11 (14%) | 4 (5%) | — | — |
| Appropriate statistical analysis used | 64 (81%) | 7 (9%) | 7 (9%) | — | — |
| **Overall: 32/79 (41%) scored ≥75%. Mean 70% (SD 20.8%; range 18–100%). 13 studies <50%, 34 50–74%, 32 ≥75%.** | | | | | |

*JBI Critical Appraisal Checklist for Cohort Studies (11 items). Percentages for Yes/No/Unclear calculated out of applicable assessments (excluding N/A). N/A = not applicable. NR = not reported.*

**Supplementary Table S5.** Risk of bias sensitivity analysis — pooled estimates for all included studies versus low risk of bias studies only (JBI score ≥75%)

| **A. Pooled timepoint survival — general surgical population** | | | | | | | |
| --- | --- | --- | --- | --- | --- | --- | --- |
|  | **All studies (n=155)** | | | **Low risk of bias only (n=68)** | | | **Δ (pp)** |
| **Timepoint** | **n** | **Pooled** | **95% CI** | **n** | **Pooled** | **95% CI** | **Δ (pp)** |
| in-hospital | 8 | 94.3% | 89.3–97.0%§ | 3 | 87.3% | 73.0–94.6%§ | -7.0 |
| 1-month | 36 | 92.4% | 90.1–94.2% | 18 | 92.4% | 88.6–95.1% | 0.0 |
| 3-month | 49 | 74.4% | 70.4–78.0% | 23 | 73.4% | 68.3–77.9% | -1.0 |
| 6-month | 61 | 64.9% | 60.3–69.2% | 24 | 64.5% | 57.3–71.1% | -0.4 |
| 1-year | 101 | 47.9% | 44.1–51.7% | 47 | 46.0% | 41.2–50.9% | -1.9 |
| 2-year | 64 | 30.8% | 25.8–36.3% | 27 | 33.8% | 23.8–45.6% | +3.0 |
| 3-year | 43 | 24.8% | 20.4–29.9% | 20 | 23.2% | 15.6–32.9% | -1.6 |
| 5-year | 29 | 16.9% | 13.1–21.4% | 14 | 14.1% | 9.9–19.9% | -2.8 |
| **Median survival** | 41 | 8.0 months | 6.0–11.0 months | 22 | 8.0 months | 6.6–10.8 months | 0 |
| **B. Pooled 1-year postoperative survival by primary tumor type** | | | | | | | |
|  | **All studies (n=155)** | | | **Low risk of bias only (n=68)** | | | **Δ (pp)** |
| **Primary tumor type** | **n** | **Pooled** | **95% CI** | **n** | **Pooled** | **95% CI** | **Δ (pp)** |
| General population | 101 | 47.7% | 43.9–51.5% | 47 | 45.9% | 41.1–50.7% | -1.8 |
| Breast | 16 | 56.4% | 46.2–66.0% | 10 | 56.8% | 43.1–69.5% | +0.4 |
| Lung | 13 | 22.7% | 12.2–38.5% | 7 | 21.8% | 5.9–55.7%§ | -0.9 |
| Renal cell | 8 | 42.0% | 23.5–63.1%§ | 5 | 46.5% | 27.9–66.2%§ | +4.5 |
| Prostate | 8 | 26.5% | 14.3–43.9%§ | 5 | 21.5% | 10.0–40.3%§ | -5.0 |
| Myeloma | 2 | 70.6% | 63.9–76.4%§ | 1 | 71.0%† | — | +0.4 |
| Thyroid | 2 | 90.0% | 90.0–90.0%§ | 1 | 90.0%† | — | 0.0 |
| Hepatocellular | 2 | 23.2% | 0.0–100.0%§ | 2 | 23.2% | 0.0–100.0%§ | 0.0 |
| **C. Secular trend in 1-year survival (weighted linear regression, weights = N, time reference = study end year)** | | | | | | | |
| **Group** | **n** | **Slope** | **95% CI; r; R²; p** | | | | **—** |
| All studies | 100 | +0.45%/yr | 0.09 to 0.81; r = 0.24; R² = 0.057; p = 0.0169 | | | | — |
| Low risk of bias only | 47 | +0.70%/yr | 0.08 to 1.33; r = 0.31; R² = 0.098; p = 0.0322 | | | | — |

*REML = restricted maximum likelihood random-effects meta-analysis on logit-transformed proportions. § REML+HKSJ = REML with Hartung-Knapp-Sidik-Jonkman correction (t-distribution, k−1 df) applied for subgroups with k<10. † Single study; no pooled estimate calculated. ‡ Prediction interval not calculable for k<3. Δ = difference in pooled estimate (low risk of bias minus all studies), in percentage points (pp). Low risk of bias defined as JBI score ≥75% of applicable items. Median survival row reports median of study-level medians; CI column shows IQR. Panel C Δ column is not applicable for the secular trend comparison.*
